# Ideal Cardiovascular Health in Former Smokers

**DOI:** 10.3390/jcm10112450

**Published:** 2021-06-01

**Authors:** Jane A. Leopold, Elliott M. Antman

**Affiliations:** 1 Division of Cardiovascular Medicine, Brigham and Women’s Hospital, Harvard Medical School, 77 Avenue Louis Pasteur, NRB630K, Boston, MA 02115, USA; 2 Division of Cardiovascular Medicine, Brigham and Women’s Hospital, Harvard Medical School, 75 Francis Street, Boston, MA 02115, USA; eantman@rics.bwh.harvard.edu

**Keywords:** smoking cessation, phenogroups, digital health devices, surveys, ideal cardiovascular health

## Abstract

Former smokers remain at increased risk for cardiovascular diseases compared to never smokers, but have lower risk than current smokers. We therefore hypothesized that former smokers would have an ideal cardiovascular health phenotype that was intermediate between current and never smokers. Differences in ideal cardiovascular health between never (*n* = 1025), former (*n* = 428), and current (*n* = 108) smokers were evaluated in the My Research Legacy study, which collected cardiovascular health data from the Life’s Simple 7 survey and digital health devices. Former smokers had a higher burden of prevalent cardiovascular disease, hypertension, diabetes mellitus, and hypercholesterolemia compared to current and never smokers (all *p* < 0.01). Former smokers’ Life’s Simple 7 Health Scores, a measure of ideal cardiovascular health, were intermediate between current and never smokers (4.9 ± 1.3 vs. 6.3 ± 1.5 vs. 7.0 ± 1.4, *p* < 0.01). As former smokers shared similarities with both current and never smokers, we performed a cluster analysis, which identified two phenogroups of former smokers. The phenogroups differed significantly across all 7 cardiovascular health and behavior categories (all *p* < 0.01). These findings suggest that former smokers are a heterogeneous group and increased attention to cardiovascular health factors and behaviors is warranted to achieve ideal cardiovascular health.

## 1. Introduction

Use of combustible tobacco is a highly preventable cause of cardiovascular morbidity and mortality worldwide [1,2,3]. Currently, 14.0% of adults report smoking tobacco daily with a higher prevalence in men than women (15.8% vs. 12.2%) [4]. In the United States, smoking prevalence was higher among individuals residing in the Midwest and South and with lower annual household incomes [5]. The relationship between smoking and cardiovascular disease risk is well known, with tobacco use recognized as an independent risk factor for incident cardiovascular disease [6]. Smoking also has a synergistic effect on the risk for cardiovascular disease when other risk factors, such as hypertension, diabetes mellitus, or hyperlipidemia are also present [5]. 

The American Heart Association incorporates smoking status into its assessment of ideal cardiovascular health in the Life’s Simple 7 survey. Life’s Simple 7 defines ideal cardiovascular health based on 7 modifiable health factors (maintaining a healthy weight, normal blood pressure, blood glucose and cholesterol levels) and health behaviors (abstinence from smoking, subscribing to a heart healthy diet, and exercise) [5,7]. The Life’s Simple 7 Health Score, a measure of ideal cardiovascular health, has been associated with both incident cardiovascular disease and adverse cardiovascular events [8,9,10]. Similarly, individuals with fewer than 5 of 7 cardiovascular health factors or health behaviors in the ideal category are also at increased risk for incident cardiovascular disease [11,12]. The importance of the Life’s Simple 7 Health Score is demonstrated by the inverse relationship between the Health Score and risk of cardiovascular events: the risk of events increases by 12% for each 1 unit decrease in the Health Score [10]. 

The prevalence of smoking has declined following the release of the United States Surgeon General’s report that outlined the health risks associated with tobacco use. Smoking rates decreased from 51% to 15.8% in men and 34% to 12.2% in women between 1965 and 2017 [4]. The cardiovascular benefits of smoking cessation include risk reduction with both short- and longer-term health effects [5]. Few studies have examined cardiovascular risk specifically in former smokers. A meta-analysis that included 15 population-based cohorts reported a summary hazard of 1.37 for cardiovascular mortality for former compared to never smokers [13]. Similarly, another study reported that former smokers had an increased risk of incident peripheral arterial disease, coronary heart disease, and stroke compared to never smokers [14]. A longitudinal analysis from the Framingham Heart Study reported an increased risk of cardiovascular disease in former smokers compared to never smokers that persisted beyond 5 years from the date of smoking cessation [15]. In the current study, we examined cardiovascular disease risk profiles in never, former, and current smokers enrolled in the My Research Legacy study, a population-based study of ideal cardiovascular health. We hypothesized that former smokers represent a heterogeneous group with variability in their ideal cardiovascular health profiles and similarities to both current and never smokers.

## 2. Materials and Methods

### 2.1. Study Cohort

The My Research Legacy study examined ideal cardiovascular health in a cross-sectional sample from across the United States that was enriched for individuals with established cardiovascular disease and cardiovascular disease in young individuals [16]. This direct-to-participant study was conducted online and sponsored by the American Heart Association. The study enrolled participants between November 2016 and October 2018. Individuals were eligible for the study if they were ≥18 years of age, resided in the United States, and had access to internet. The study was approved by the Advarra Institutional Review Board (www.advarra.com, accessed on 22 March 2021) (approval# 31995) and eligible individuals signed informed consent online. Participants self-reported baseline demographic data, history of cardiovascular diseases, cardiovascular disease risk factors, and completed the Life’s Simple 7 survey of ideal cardiovascular health [16].

Use of a digital health device was not mandated by the study. A subset of individuals that provided informed consent for this part of the study either received a Fitbit Charge 2 digital health and activity device or registered their own device with the study. Individuals with digital scales linked their scales to their other digital health devices. Participants with digital devices were provided with a unique link to Validic (Validic Inc., Durham, NC, USA) to upload digital health device data. Uploaded data were de-identified and categorized as weight, fitness or routine data by Validic based on device-specific algorithms. De-identified data were transmitted to secure servers maintained by The Broad Institute (Cambridge, MA, USA) and REAN Cloud LLC (Herndon, VA, USA) [16].

### 2.2. Smoking Status and Ideal Cardiovascular Health

Smoking status was ascertained as current, quit ≤ 12 months ago, quit > 12 months ago, or never by participant self-report. Former smoker was defined as a combination of the quit ≤ 12 months ago and quit > 12 months ago categories. Ideal cardiovascular health was evaluated using the Life’s Simple 7 survey instrument. All participants answered the online survey questions about health factors (blood pressure, blood glucose and cholesterol levels, and weight) as well as health behaviors (tobacco use, diet, and exercise). The Life’s Simple 7 survey assigns a score of 0, 1, or 2 corresponding to poor, intermediate, or ideal for each of the 7 health factors and health behaviors based on criteria defined by a panel of experts. The Life’s Simple 7 Health Score is calculated using the health categories scores and ranges between 0 (poor) and 10 (ideal) [7,17]. Ideal cardiovascular health was defined by having an ideal score for a minimum of 5 categories or a Life’s Simple 7 Health Score of >7.0 [5,18]. 

### 2.3. Cluster Analysis

In order to explore heterogeneity among former smokers, a factor analysis of mixed data was performed to reduce the dimensionality of the data [19]. Dissimilarity among former smokers was calculated using Gower distance [20]. To calculate Gower distance, binary categorical variables with asymmetric properties and ordinal variables were labeled accordingly. Silhouette width was utilized to determine the optimal number of clusters and a partitioning around mediods algorithm was used to identify clusters [21]. Phenogroups were defined by cluster assignment and visualized using t-distributed stochastic neighborhood embedding. Cluster analysis and cluster visualization were performed using R (https:r-project.org, accessed on 31 May 2021; version 3.6.2) and the FactoMineR, factoextra, and Rtsne packages.

### 2.4. Statistical Analysis

Sample size to ensure that former smokers were represented adequately in our study population was calculated based on the study sample size of 1561 participants who provided Life’s Simple 7 survey data. With a prevalence of former smokers of 20.9% [4], to achieve 95% power with an alpha = 0.05, the minimum sample size of former smokers required was 219 participants. For current smokers with a prevalence of 14%, to achieve 85% power with an alpha = 0.05, the minimum sample size is 94 participants. For never smokers with a prevalence of 65.1% [4], to achieve 95% power with an alpha = 0.05, the minimum sample size is 286 participants.

Comparisons between continuous variables were done using *t*-tests or one-way ANOVA. The chi-square test or Fisher’s exact test was used for comparisons between categorical variables. Nonparametric data were analyzed using the Wilcoxon-rank sum test or Kruskal–Wallis test. Data are presented as mean ± SD and *p*-values < 0.05 were considered significant. Data were analyzed using Stata 15/SE 15.1 (StataCorp LLC, College Station, TX, USA) and Prism 9.0 (GraphPad, San Diego, CA, USA).

## 3. Results

### 3.1. Differences in Ideal Cardiovascular Health Data among Never, Former, and Current Smokers

Of the 1561 participants who completed an assessment of ideal cardiovascular health, 1025 were never smokers, 428 were former smokers, and 108 were current smokers. There were differences in age among never, former, and current smokers (43.4 ± 13.7 vs. 46.9 ± 11.5 vs. 41.0 ± 10.1 yrs, *p* < 0.01 by ANOVA) with former smokers being older than the other groups (Table 1). While there was no difference between the groups with respect to gender distribution or regionality, there were differences in race and ethnicity with current and former smokers having a higher percentage of white participants and a lower percentage of Asian, black, or Hispanic participants than never smokers (*p* < 0.02). There was also a difference in the prevalence of prior cardiovascular diseases (*p* < 0.01), diabetes mellitus (*p* < 0.04), hypertension (*p* < 0.01), and hypercholesterolemia (*p* < 0.01) between the groups with former smokers having a higher prevalence of prior cardiovascular disease and associated risk factors. 

**Figure 1:**
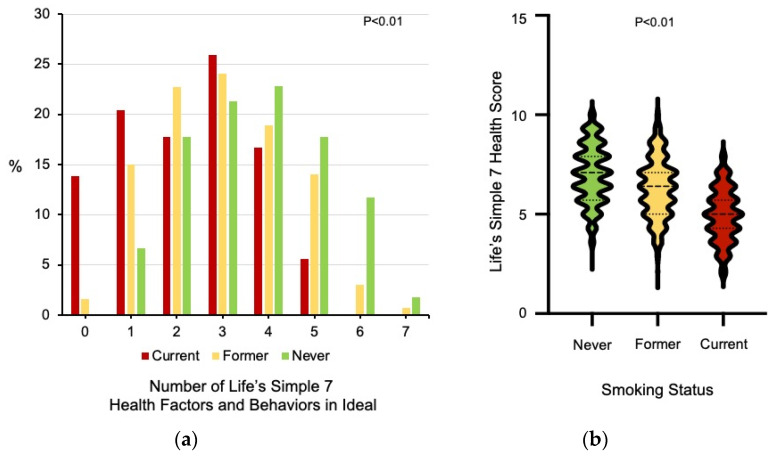
Life’s Simple 7 health factors and behaviors in the ideal range and ideal cardiovascular health. (**a**) The 7 health factors and health behaviors that are assessed by the Life’s Simple 7 survey instrument are scored as ideal, intermediate, or poor according to criteria defined by an expert panel [7,17]. The number of categories scored as ideal is presented for current (*n* = 108), former (*n* = 428), and never smokers (*n* = 1025). Data was analyzed using the Kruskal–Wallis test. (**b**) The distribution of Life’s Simple 7 Health Scores for never, former, and current smokers is shown as violin plots with median and quartiles identified as dashed lines. Data was analyzed using the Kruskal–Wallis test.

There were also differences between the groups with respect to health factors, including weight and body mass index (BMI), systolic blood pressure, total cholesterol and blood glucose levels (all *p* < 0.01) with former smokers having higher weights and BMIs as well as blood glucose levels compared to never and current smokers (Table 1). The groups also differed significantly in cardiovascular health behaviors, such as heart healthy dietary choices and exercise. There were differences in vegetable (*p* < 0.01), fruit (*p* < 0.01), fish (*p* < 0.02), whole grain consumption (*p* < 0.01), and sugar-sweetened beverage consumption (2.1 ± 3.1 vs. 2.3 ± 3.2 vs. 5.4 ± 4.8 drinks/week, *p* < 0.01 by ANOVA) between current, former, and never smokers. There was also a significant difference in the weekly minutes of vigorous exercise with current smokers exercising fewer minutes than former and never smokers (*p* < 0.01). The percentage of individuals with ideal scores in 5 or more Life’s Simple 7 health factors and behaviors differed significantly between the groups, with former smokers having an intermediate profile between current and never smokers (Figure 1a). Similarly, there were differences in Life’s Simple 7 Health Scores between the smoking groups with former smokers having and intermediate score between current and never smokers (7.0 ± 1.4 vs. 6.3 ± 1.5 vs. 4.9 ± 1.3 *p* < 0.01 by Kruskal–Wallis test) (Figure 1b).

We also compared differences between former smokers who quit combustible tobacco use ≤ 12 months prior to study entry (*n* = 59) versus those that quit > 12 months prior (*n* = 369). Individuals that quit tobacco use ≤ 12 months before study entry were younger (42.6 ± 9.1 vs. 47.6 ± 11.7 yrs, *p* < 0.01), were more likely to have cardiovascular disease (64.4% vs. 47.7%, *p* < 0.02), performed fewer weekly minutes of moderate exercise (157.8 ± 158.1 vs. 227.3 ± 244.8 min, *p* < 0.04), and were less likely to have 5 or more Life’s Simple 7 health factors and behaviors in the ideal range (6.78% vs. 19.5%, *p* < 0.01) than individuals who quit smoking > 12 months before study entry. There were no differences between the groups with respect to gender, race or ethnicity and region; weight or BMI; blood pressure, cholesterol levels, diabetes mellitus or use of medications for these risk factors; weekly minutes of vigorous exercise; or dietary habits. 

### 3.2. Heterogeneity among Former Smokers

**Figure 2:**
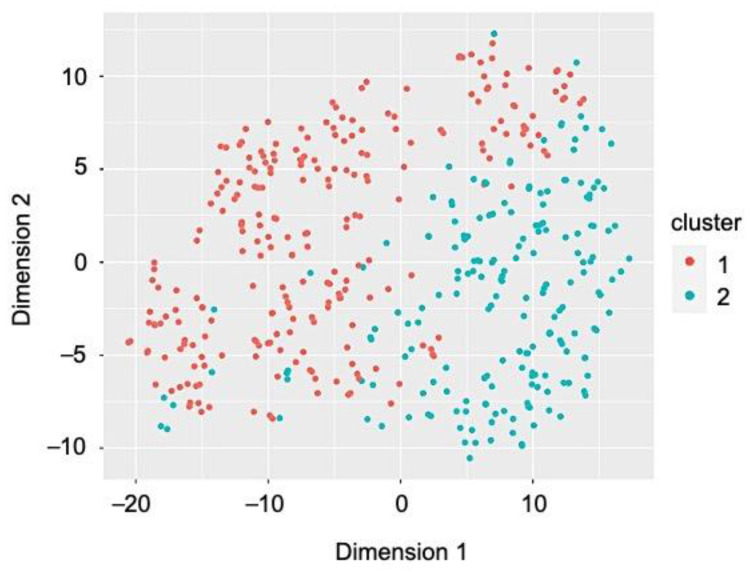
Cluster analysis identified two phenogroups of former smokers. In order to identify phenogroups within the population of former smokers, Gower distance to identify dissimilarity between individuals was calculated and a partitioning around mediods algorithm was used to identify the clusters. The clusters were visualized using t-distributed stochastic neighborhood embedding to reduce dimensionality of the data.

Next, we explored heterogeneity among former smokers (*n* = 428) as we observed that they shared some cardiovascular health factors and health behaviors with the current smokers (weight and BMI, blood pressure, cholesterol levels) and others with never smokers (diet). In order to determine if there were clusters or phenogroups of former smokers, we performed a factor analysis of mixed data. This demonstrated that the first two dimensions explained 23.7% of the variability among former smokers. Silhouette width determined that there were two clusters within the population of former smokers and a partitioning around mediods algorithm identified the phenogroups (Figure 2). This analysis resulted in 241 individuals assigned to phenogroup 1 and 187 individuals assigned to phenogroup 2. There was no difference between the phenogroups in the percentage of former smokers who stopped using combustible tobacco for greater than one year at the time of study entry (83.4% vs. 89.8%, *p* = 0.06).

Former smokers in phenogroup 1 were older than those in phenogroup 2 (49.7 ± 11.1 vs. 43.4 ± 11.2 yrs, *p* < 0.01 by ANOVA), but there were no differences between the phenogroups with respect to gender distribution, race and ethnicity, or region (Table 2). 

**Figure 3:**
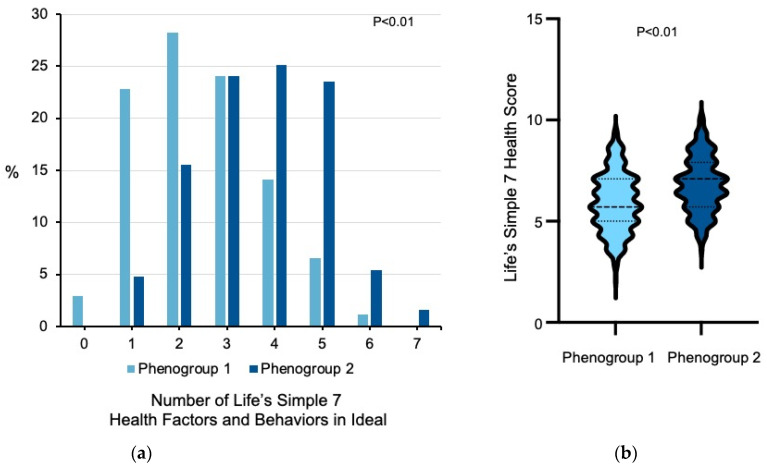
Former smoker phenogroups and measures of ideal cardiovascular health. (**a**) Life’s Simple 7 health factors and health behaviors are scored according to predefined criteria as poor, intermediate, or ideal. For each former smoker in phenogroup 1 (*n* = 241) and phenogroup 2 (*n* = 187), the number of health factors or behaviors that were scored in the ideal category is shown. Data was analyzed using the Wilcoxon rank sum test. (**b**) The distribution of Life’s Simple 7 Health Scores for former smokers in each of the phenogroups is presented as violin plots. The median and quartiles are denoted by dashed lines. Data was analyzed using the Wilcoxon rank sum test.

Former smokers in phenogroup 1 had a significantly higher prevalence of prior cardiovascular disease, hypertension, hypercholesterolemia, diabetes mellitus, and corresponding medication use compared to participants in phenogroup 2 (all *p* < 0.01). Participants in phenogroup 1 had higher weight and BMI (*p* < 0.02), systolic and diastolic blood pressures (*p* < 0.03), and blood glucose levels (*p* < 0.01) compared to participants in phenogroup 2. Despite their adverse cardiovascular risk factor profile, individuals in phenogroup 1 subscribed to more heart healthy dietary choices and behaviors than individuals in phenogroup 2, with higher consumption of fruit (*p* < 0.02) and fish (*p* < 0.03) and lower consumption of sugar-sweetened beverages (*p* < 0.01). Compared to phenogroup 2, individuals in phenogroup 1 also tended to avoid prepacked foods, eating out, and added salt (all *p* < 0.01). Although participants in the phenogroups performed a similar number of minutes per week of moderate exercise, individuals in phenogroup 1 completed fewer minutes per week of vigorous exercise than participants in phenogroup 2 (*p* < 0.01). Individuals in phenogroup 1 had fewer individuals with 5 or more Life’s Simple 7 health factors and behaviors in the ideal category than individuals in phenogroup 2 (7.9% vs. 30.5%, *p* < 0.01) and lower Life’s Simple 7 Health Scores (5.9 ± 1.4 vs. 6.8 ± 1.3, *p* < 0.01 by Wilcoxon rank sum test) (Figure 3). Taken together, these data indicate that there is heterogeneity among former smokers in the study.

### 3.3. Digital Health Data and Life’s Simple 7 Health Score

We next sought to determine how objective data from digital health devices data influenced assessment of ideal cardiovascular health among never, former and current smokers. Of the 390 individuals who transmitted digital health device data, only 6 were current smokers and of the 6, only 3 transmitted data. Therefore, we analyzed digital health data for never smokers (*n* = 278) and former smokers (*n* = 106) only. Of the remaining 384 individuals, 95 participants did not transmit digital weight data and 35 did not transmit digital exercise data. There were no differences in types of digital health devices registered with the study, with the majority of participants in each group using a Fitbit device (*p =* 0.38). There was also no difference between never and former smokers with respect to average daily step count (7723.1 ± 4465.8 vs. 7896.2 ± 4776.3, *p =* 0.75), suggesting that participants in both groups utilized their digital devices similarly.

Comparable to self-reported data, digital health device-measured weight (78.9 ± 19.4 kg vs. 85.7 ± 22.9, *p* < 0.01) and BMI (27.8 ± 6.3 vs. 30.4 ± 8.2 kg/m^2^, *p* < 0.01) was significantly higher in former smokers as compared to never smokers (Table 3). There was also a significant difference in the distribution of poor, intermediate, and ideal weight scores based on digital health device data between former and never smokers (*p* < 0.04). Digital health device-recorded activity was examined over a 7-day time period. Compared to never smokers, former smokers recorded fewer weekly minutes of moderate exercise (149.9 ± 190.8 vs. 98.3 ± 117.4 min/week, *p* < 0.02), but similar weekly minutes of vigorous exercise (165.8 ± 230.9 vs.161.4 ± 227.9 min/week, *p =* 0.87). This resulted in a similar distribution of nonsmokers and former smokers with poor, intermediate, and ideal activity scores (*p =* 0.06). When the Life’s Simple 7 Health Score was calculated using digital health weight and activity data, the Health Score remained lower in former smokers than never smokers (7.2 ± 1.3 vs. 6.5 ± 1.3, *p* < 0.01 by Wilcoxon rank sum test).

## 4. Discussion

In the current study, we examined ideal cardiovascular health using the American Heart Association’s Life’s Simple 7 survey instrument to examine cardiovascular health factor and health behavior patterns among former smokers as compared to current and never smokers. We found that similar to current smokers, former smokers had a higher burden of cardiovascular risk factors and established cardiovascular disease than never smokers. Among the modifiable health factors, former smokers had a higher weight and BMI than the never or current smokers; a similar systolic and diastolic blood pressure; and, total cholesterol levels similar to current smokers. These differences were apparent despite a higher incidence of medication use for hypertension and hypercholesterolemia than either never or current smokers. In contrast, former smokers more closely resembled never smokers in terms of dietary choices and habits and subscribed to a heart healthy diet. The exercise profiles of former smokers, however, was intermediate between current and never smokers. This resulted in former smokers having an ideal cardiovascular Health Score that was intermediate between current and never smokers. When incorporating data from digital health devices, we found that former smokers did more weekly minutes of vigorous exercise than they reported on the Life’s Simple 7 survey instrument. However, owing to higher weight and BMI as well as differences in other health factors and behaviors, former smokers still had lower overall Life’s Simple 7 Health Scores than never smokers. 

A second finding from our study was that there was heterogeneity among the former smokers with the emergence of two phenogroups: one phenogroup that aligned with current smokers’ cardiovascular health factors and health behaviors profile while the other phenogroup more closely resembled the never smokers. There were significant differences between the phenogroups with respect to health factors, including the prevalence of diabetes mellitus, hypertension, and hypercholesterolemia, and weight as well as health behaviors, including diet and exercise. Individuals in phenogroup 1 had a lower prevalence of ideal scores across all 7 health factors and health behaviors categories in the Life’s Simple 7 survey assessment of ideal cardiovascular health than individuals in phenogroup 2. Therefore, it appears that participants in phenogroup 2 had taken steps to optimize all cardiovascular disease risk factors and health behaviors while individuals in phenogroup 1 would benefit from additional interventions to achieve ideal cardiovascular health.

The health benefits of smoking cessation are numerous and smoking cessation has been shown to decrease the risk of major adverse cardiovascular events, including mortality, for individuals with or without established disease [5]. In the Atherosclerosis Risk in Communities (ARIC) Study, investigators reported that former smokers retained a significantly elevated risk for coronary heart disease for 20 years after the time of smoking cessation as well as a prolonged risk for peripheral arterial disease for up to 30 years [14]. In the Framingham Heart Study, former smokers also had an increased risk of incident cardiovascular disease compared to never smokers, which declined in a time-dependent manner and reached parity after 25 years of smoking cessation [15]. Former smokers in the ARIC Study tended towards a phenotype that was intermediate between current and never smokers, with the exception of higher mean age, level of physical activity, and medication use for hypercholesterolemia. Recognition of this intermediate phenotype also lends support to the concept of heterogeneity among former smokers, which we explored further in our study. In contrast to the ARIC Study, we did not exclude individuals with prevalent cardiovascular diseases. We found that former smokers had a higher prevalence of established cardiovascular diseases, hypertension, hypercholesterolemia, and diabetes mellitus than current or never smokers. Our study also collected dietary information and exercise data on a more granular level than the ARIC Study, which allowed us to assess ideal cardiovascular health as well as identify areas where further risk factor and behavioral modification would improve ideal cardiovascular health. 

Former smokers have been shown to benefit from maintaining a healthy weight, a heart healthy diet, and an exercise program with significant reductions in recurrent cardiovascular events in individuals with prevalent cardiovascular disease [22]. The relationship between smoking cessation, weight, and diet is complex. Smoking cessation is known to be associated with weight gain with women in the United States gaining an average of 3.8 kg while men gain ~2.8 kg. Other studies have reported higher weight gains. In the Framingham Heart Study, individuals gained an average of 5.8 ± 0.8 kg after smoking cessation and other studies have shown that in up to 20–40% of individuals, the average weight gain may be ≥13 kg [23,24,25]. Differences in age, diet, and physical activity were among the reasons cited for weight gain in former smokers [23], suggesting that a focus on diet and exercise would be beneficial. Similar to what we observed, studies have reported that former smokers were more likely than current smokers to adhere to a heart healthy diet, such as the Mediterranean diet [26]. Investigators have also found that former smokers were more likely to increase their exercise habits after smoking cessation, which is in line with achieving ideal cardiovascular health [27]. Former smokers who added physical activity to their daily routine also reported enhanced health-related quality of life [28]. 

Our study has some limitations that may influence the generalizability of our findings. First, while our study enrolled both current and former smokers, we did not collect data on daily smoking dose. We are also unable to provide more detail on the duration of smoking cessation beyond a one year cutoff as the Life’s Simple 7 survey instrument does not ask for these data. Since our study had a fixed sample size and enrolled a predominantly white cohort and fewer individuals of other races and ethnicities, it is possible that our results may be different if we enrolled a larger sample and may be less generalizable to other racial and ethnic groups. Former smokers in our study were also older than never or current smokers and some differences between the groups may have been age dependent. Since registration of a digital health device was not mandatory to participate in our study, we enrolled only a few current smokers who provided digital health device data. Thus, we were unable to compare weight and activity data acquired by digital health devices from smokers to former and never smokers. Nevertheless, among participants that had digital device data available for analysis, we found that digital devices, which provided an objective measure of activity, recorded fewer minutes of moderate activity and more minutes of vigorous activity than participants reported. This has implications for assessments of activity levels in former smokers and ideal cardiovascular health. 

## 5. Conclusions

Abstinence from smoking is an integral component of ideal cardiovascular health. Although the Life’s Simple 7 assessment of ideal cardiovascular health considers smoking cessation for >12 months as ideal, studies have shown that the risk of cardiovascular diseases remains elevated for a prolonged time period in former smokers compared to never smokers [14,15]. This suggests that former smokers would benefit from optimization of cardiovascular health factors and health behaviors. Owing to the heterogeneity among former smokers identified in our study, with one phenogroup having risk factor profiles and health behaviors akin to current smokers while the other was closer to never smokers, strategies to achieve ideal cardiovascular health in this at-risk group will require a personalized risk assessment. Furthermore, our data suggest that despite more than one year of combustible tobacco abstinence, the distinction between the phenogroups persists. Future studies are needed to determine whether or not phenogroup assignment has implications for cardiovascular disease risk and if interventions modify this risk or the distinction between phenogroups remains. Taken together, in addition to promoting smoking cessation in current smokers, our study suggests that increased attention to cardiovascular health factors and health habits in former smokers is warranted to achieve ideal cardiovascular health. 

## Figures and Tables

**Table 1:** Participant self-reported demographics and Life’s Simple 7 data by smoking status.

	Never Smoker (*n* = 1025)	Former Smoker (*n* = 428)		Current Smoker (*n* = 108)	*p*-Value
Age (yrs)	43.4 ± 13.7	46.9 ± 11.5		41.0 ± 10.1	<0.01
Gender (% female)	79.8	79.7		85.2	0.40
Race and Ethnicity (no.)					<0.02
Asian	37	4		1	
Black	46	11		3	
Hispanic	46	20		2	
White	868	372		97	
Other	28	21		5	
Region (no.)					0.71
Northeast	145	65		17	
South	400	182		40	
Midwest	258	96		24	
West	222	85		27	
Diagnosed with Cardiovascular Disease (%)	29.6	50.0		45.4	<0.01
Diabetes mellitus (%)	8.9	13.3		12.0	<0.04
Hypertension (%)	46.1	59.1		49.1	<0.01
Hypercholesterolemia (%)	49.5	63.6		50.9	<0.01
Medications (%)					
Diabetes mellitus	7.8	11.2		8.3	0.11
Hypertension	27.3	45.3		30.6	<0.01
Hypercholesterolemia	16.9	30.1		17.6	<0.01
CLINICAL DATA					
Weight (kg)	81.7 ± 23.1	89.7 ± 26.1		86.5 ± 25.6	<0.01
BMI (kg/m^2^)	29.1 ± 7.9	31.7 ± 8.7		31.3 ± 8.0	<0.01
Systolic blood pressure (mmHg) *	117.1 ± 12.0	120.1 ± 14.1		119.3 ± 12.9	<0.01
Diastolic blood pressure (mmHg) *	73.2 ± 8.5	74.0 ± 9.1		72.6 ± 8.0	0.13
Total cholesterol (mmol/L) *	187 ± 29	192 ± 29		193 ± 27	<0.01
Blood glucose (mmol/L) *	98.0 ± 17.0	102.0 ± 22.0		99.0 ± 16.0	<0.01
DIET					
Vegetables/day (cups)	1.9 ± 1.3	1.9 ± 1.3		1.4 ± 1.3	<0.01
Fruit/day (cups)	1.4 ± 1.1	1.2 ± 1.0		1.0 ± 1.1	<0.01
Fish (servings/week)	1.0 ± 1.0	0.9 ± 1.0		0.7 ± 0.9	<0.02
Whole grains (servings/day)	1.7 ± 1.2	1.5 ± 1.2		1.4 ± 1.1	<0.01
Sugar-sweetened beverages (servings/week)	2.1 ± 3.1	2.3 ± 3.2		5.4 ± 4.8	<0.01
Avoid prepackaged foods (%)	52.7	54.0		40.7	<0.05
Avoid eating out (%)	37.0	41.4		28.7	<0.05
Avoid salt at home (%)	55.9	59.1		53.7	0.43
EXERCISE					
Moderate exercise (min/week)	195.5 ± 200.5	217.9 ± 235.8	229.9 ± 255.7		0.08
Vigorous exercise (min/week)	77.2 ± 121.5	52.3 ± 107.2	33.2 ± 94.5		<0.01
LIFE’S SIMPLE 7					
Smoking score (%)					<0.01
Poor	0	0	100		
Intermediate	0	13.8	0		
Ideal	100	86.2	0		
Activity score (%)					<0.02
Poor	1.5	2.8	2.8		
Intermediate	35.8	39.3	49.1		
Ideal	62.7	57.9	48.1		
Diet score (%)					<0.01
Poor	41.5	45.3	64.8		
Intermediate	49.4	45.1	33.3		
Ideal	9.1	9.6	1.9		
Weight score (%)					<0.01
Poor	37.7	50.2	54.6		
Intermediate	25.0	23.8	18.5		
Ideal	37.3	26.0	26.9		
Blood glucose score (%)					<0.01
Poor	2.9	5.8	4.6		
Intermediate	33.8	40.4	36.1		
Ideal	63.3	53.7	59.3		
Cholesterol score (%)					<0.01
Poor	2.1	3.7	1.9		
Intermediate	46.0	59.6	48.1		
Ideal	51.9	36.7	50.0		
Blood pressure score (%)					<0.01
Poor	4.8	9.4	6.5		
Intermediate	49.8	59.3	51.9		
Ideal	45.4	31.3	41.6		
LS7 Health Score	7.0 ± 1.4	6.3 ± 1.5	4.9 ± 1.3		<0.01

* Contains imputed data from Life’s Simple 7, Categorical variables are analyzed by Chi-Square test, Continuous variables are analyzed by ANOVA, Non-parametric variables were analyzed by Kruskal-Wallis test.

**Table 2:** Former smoker self-reported demographics and ideal cardiovascular health data.

	Phenogroup 1 (*n* = 241)	Phenogroup 2 (*n* = 187)	*p*-Value
Age (yrs)	49.7 ± 11.1	43.4 ± 11.2	<0.01
Gender (% female)	77.6	82.4	0.23
Race and Ethnicity (no.)			0.49
Asian	3	1	
Black	7	4	
Hispanic	9	11	
White	213	159	
Other	9	12	
Region (no.)			0.59
Northeast	38	27	
South	102	80	
Midwest	58	38	
West	43	42	
Diagnosed with Cardiovascular Disease (%)	72.2	21.4	<0.01
Diabetes mellitus (%)	19.2	4.81	<0.01
Hypertension (%)	82.2	29.4	<0.01
Hypercholesterolemia (%)	80.9	41.2	<0.01
Medications (%)			
Diabetes mellitus	17.0	3.7	<0.01
Hypertension	74.2	8.0	<0.01
Hypercholesterolemia	46.5	9.1	<0.01
CLINICAL DATA			
Weight (kg)	92.3 ± 26.8	86.2 ± 24.8	<0.02
BMI (kg/m^2^)	32.6 ± 9.0	30.5 ± 8.2	<0.02
Systolic blood pressure (mmHg) *	122.2 ± 14.9	117.4 ± 12.5	<0.01
Diastolic blood pressure (mmHg) *	74.9 ± 10.0	72.9 ± 7.8	<0.03
Total cholesterol (mmol/L) *	190.6 ± 31.6	192.8 ± 26.6	0.45
Blood glucose (mmol/L) *	104.9 ± 22.7	99.2 ± 19.8	<0.01
DIET			
Vegetables/day (cups)	1.9 ± 1.3	1.9 ± 1.3	0.80
Fruit/day (cups)	1.4 ± 1.1	1.1 ± 0.8	<0.02
Fish (servings/week)	1.0 ± 1.0	0.8 ± 0.9	<0.03
Whole grains (servings/day)	1.5 ± 1.2	1.5 ± 1.2	0.92
Sugar-sweetened beverages (servings/week)	1.9 ± 2.9	2.8 ± 3.6	<0.01
Avoid prepackaged foods (%)	65.6	39.0	<0.01
Avoid eating out (%)	56.0	22.5	<0.01
Avoid salt at home (%)	78.0	34.8	<0.01
EXERCISE			
Moderate exercise (min/week)	200.4 ± 217.1	240.5 ± 256.7	0.08
Vigorous exercise (min/week)	43.0 ± 92.7	64.2 ± 122.6	<0.05
LIFE’S SIMPLE 7			
Smoking score (%)			<0.06
Poor	0	0	
Intermediate	16.6	10.2	
Ideal	83.4	89.9	
Activity score (%)			0.24
Poor	3.3	2.1	
Intermediate	41.1	36.9	
Ideal	55.6	61.0	
Diet score (%)			<0.01
Poor	34.9	58.8	
Intermediate	51.9	36.4	
Ideal	13.3	4.8	
Weight score (%)			<0.01
Poor	55.6	43.3	
Intermediate	23.7	24.1	
Ideal	20.7	32.6	
Blood glucose score (%)			<0.01
Poor	8.7	2.1	
Intermediate	49.0	29.4	
Ideal	42.3	68.5	
Cholesterol score (%)			<0.01
Poor	3.7	3.7	
Intermediate	77.2	36.9	
Ideal	19.1	59.4	
Blood pressure score (%)			<0.01
Poor	12.9	4.8	
Intermediate	73.0	41.7	
Ideal	14.1	53.5	
LS7 Health Score	5.9 ± 1.4	6.8 ± 1.3	<0.01

* Contains imputed data from Life’s Simple 7, Categorical variables are analyzed by Chi-Square test, Continuous variables are analyzed by *t*-test, Non-parametric variables were analyzed by Wilcoxon rank sum test.

**Table 3:** Participant digital health device weight and exercise data *.

	**Never Smokers** **(*n* = 269)**	**Former Smokers** **(*n* = 101)**	***p*-Value**
Steps per day	7896.2 ± 4776.3	7723.1 ± 4465.8	0.75
	**Never Smokers** **(*n* = 204)**	**Former Smokers** **(*n* = 85)**	***p*-Value**
Weight (kg)	78.9 ± 19.4	85.7 ± 22.9	<0.01
Absolute change in weight (kg) Reported vs. Measured	−0.8 ± 3.9	0.01 ± 5.2	0.25
BMI (kg/m^2^)	27.8 ± 6.3	30.4 ± 8.1	<0.01
Absolute change in BMI (kg/m^2^) Reported vs. Measured	−0.3 ± 1.4	0.1 ± 2.0	0.27
Healthy Weight Score (%)			<0.04
Poor	30.9	44.7	
Intermediate	31.4	27.1	
Ideal	37.7	28.2	
	**Never Smokers** **(*n* = 259)**	**Former Smokers** **(*n* = 93)**	***p*-Value**
Moderate exercise (min/week)	149.9 ± 190.8	98.3 ± 117.4	<0.02
Absolute change in moderate exercise (min/week) Reported vs. Measured	54.5 ± 250.6	146.5 ± 244.6	<0.01
Vigorous exercise (min/week)	165.8 ± 230.9	161.4 ± 227.9	0.87
Absolute change in vigorous exercise (min/week) Reported vs. Measured	−70.4 ± 233.6	−92.7 ± 201.8	0.41
Physical Activity Score (%)			0.06
Poor	0.0	0.0	
Intermediate	36.3	47.3	
Ideal	63.7	52.7	
	**Never Smokers** **(*n* = 193)**	**Former Smokers** **(*n* = 77)**	***p*-Value**
Recalculated			
LS7 Health Score	7.2 ± 1.3	6.5 ± 1.3	<0.01

* A total of 390 participants contributed digital health device data: step counts were available from 370 participants; weight data was available from 289 participants; and, exercise data was available from 352 participants. Of these participants, a total of 207 participants had both weight and exercise data available.

## Data Availability

For information regarding data availability, please contact the corresponding author. The data are not publicly available due to privacy restrictions and protection of personal data.
